# Challenges and Opportunities for Theory-Grounded Data Visualization in Extended Reality

**DOI:** 10.1109/MCG.2026.3685140

**Published:** 2026

**Authors:** Andreas Bueckle, Christiane V. R. Hütter, Renata G. Raidou, Chris Bryan, Peter Mindek, Hannes Kaufmann, Niklas Elmqvist, Jörg Menche, Katy Börner

**Affiliations:** Indiana University, Bloomington, IN, 47408, USA; Vienna BioCenter PhD Program, a Doctoral School of the University of Vienna and the Medical University of Vienna, 1030, Vienna, Austria; Vienna University of Technology (TU Wien), 1040, Vienna, Austria; Arizona State University, Tempe, AZ, 85281, USA; Nanographics GmbH, 1030, Vienna, Austria; Vienna University of Technology (TU Wien), 1040, Vienna, Austria; Aarhus University, 8200, Aarhus, Denmark; The Ludwig Boltzmann Institute for Network Medicine, University of Vienna, 1090, Vienna, Austria; Indiana University, Bloomington, IN, 47408, USA and also Canadian Institute for Advanced Research, Toronto, ON, M5G 1M1, Canada

## Abstract

Numerous frameworks have been developed for creating, interpreting, and teaching data visualizations. The past decade has seen advances in visualization paradigms beyond keyboard and mouse (e.g., immersive analytics). While mature visualization libraries exist for screen-based technologies, no comparable counterparts have emerged for immersive visualization due to scattered platforms, while theoretical frameworks require inclusion of extended reality (XR) and 3-D visualizations tailored to embodied exploration. Here, we synthesize challenges and opportunities for theory-grounded data visualization in XR to examine how established principles translate and expand to immersive environments. This work supports the development of both theory and practical tools for principled data visualization and exploration using XR in support of real-world applications.

The past decade has seen significant advances in visualization paradigms beyond windows, icons, menus, pointers (WIMP) user interfaces (UIs) for data visualization, such as *immersive analytics*, driven by the ultimate need for user-centric novel approaches to generate interpretable, interactive data representations for exploration in various domains while dealing with diverse data modalities and dimensions.^1^ Data exploration can strongly benefit from collaboration, fostering simultaneously sharing insights between researchers while interacting on the very same data in a common (virtual) space, without being limited by the need of physical proximity.^2^ These user-focused challenges for intuitive data exploration coincided with the increasing availability of affordable, portable, off-the-shelf extended reality (*XR*) *devices*, including virtual, augmented, and mixed reality hardware (VR, AR, and MR). While interpretability and explorability of datasets within *immersive environments* are certainly attached to an end user, the technologies and *frameworks* needed to build these in the first place are have to be usable by developers and researchers. Existing theoretical *frameworks* and practical tools remain inadequate for capturing the unique affordances of *immersive environments*, which, unlike *desktop environments*, fundamentally alter how users perceive scale, spatial relationships, *embodiment*, and interaction within data visualizations.^3^ Concepts, such as physical navigation, collaboration, and multimodal input, introduce design dimensions that are not addressed by traditional 2-D or 3-D visualization theories for *desktop environments*. As a result, *XR* visualizations often rely on repurposed metaphors from screen-based systems rather than systematic, theory-driven guidance. Similarly, while 2-D (and, to some extent, 3-D) visualizations benefit from mature *visualization libraries*, the *XR* landscape lacks equivalent standardized approaches. Current immersive visualization development is fragmented across *platforms*, such as Unity,^a^ Unreal Engine,^b^ and WebXR,^c^ each requiring specialized expertise while offering limited theoretical grounding for *visualization libraries* and design decisions to consistently follow in a *platform*-independent *framework*. These *platforms* are broadly separated into two major categories: *(native) game engines* and web-based *platforms*, see this overview for a comparison of key features GitHub,^d^ Zenodo/all Supplemental Tables,^e^ Zenodo/direct preview).^f^ This fragmentation not only raises the technical barrier for researchers and practitioners but also hinders reproducibility, comparability, and cumulative knowledge building across studies. Without shared abstractions, concepts, and pipelines, insights gained from individual *XR visualization libraries* remain difficult to generalize or transfer across *platforms*, domains, or target groups.

This Viewpoint provides a retrospective on major insights from our IEEE VIS 2025 panel titled “TALK, TRY, TELL: Challenges and Opportunities for Theory-Grounded Data Visualization in Extended Reality” with a “*TALK, TRY, TELL*” format that combined theoretical discourse with hands-on experiences. During the panel, five experts presented their own research on data visualization beyond 2-D screens (*TALK*), followed by hands-on testing (*TRY*), and finishing with an audience feedback session (*TELL*). To enable unified terminology, Table 1 lists key terminology for *XR* visualizations. These key terms are rendered in *italics* whenever used in this article. A companion website with photos from the panel is available.

## Existing Work: 2-D Visualization Libraries

Mature 2-D *visualization libraries* exist across programming languages and neatly integrate with software packages for data analysis. Three major programming languages and their *visualization libraries* are briefly discussed here. This is not a complete list but exemplifies features of a mature landscape for *visualization libraries*.

*Base R* has built-in graphics with core plots, such as histograms and scatter graphs. Building on this, the tidyverse, specifically *ggplot2*, enables advanced data visualization. Other *visualization libraries* address specialized needs for data visualization (e.g., *ggrepel* to handle overlapping text labels). With Shiny, these visualizations can be deployed to the web.

*Python* provides a large array of *visualization libraries* that neatly mesh with its overall status as a multipurpose programming language for data analysis. *matplotlib* and *seaborn* serve as foundational plotting *libraries*. Interactive data visualizations are available via *Bokeh, Altair*, and *plotly*. Scientific and 3-D visualization are made possible by *napari*; networks can be built with *networkX* and *pyvis*.

Finally, *JavaScript* offers mature *visualization libraries* for the web. *D3, Vega*, and *Vega-Lite* form a stack of increasingly abstract technologies that enable data visualization for standard and specialized 2-D visualization types. 3-D visualizations are possible with Three.js, Deck.gl, and Babylon.js, all of which are WebGL-based.

While these data *visualization libraries* cover a wide range of visualization types, data types, reference systems, and deployment methods, they share some core features:

### Shared pipeline:

They implement a basic pipeline of Data—Transformations—Scales—Layout—Graphic symbols/variables—Deployment—Interaction, a sequence also captured by the Data Visualization Literacy Framework Process Model.^6^ Usually, they achieve this by implementing an underlying *grammar of graphics*. One of the most prominent examples is *ggplot2* as it implements the “Grammar of Graphics” by Wilkinson.^5^ They are able to perform various data transformations (e.g., logarithmic scaling). Most can determine the data scale of a variable on their own with minimal input and will raise errors if needed. Finally, they map data records to visual properties in charts, graphs, maps, or networks.

### Rendering and appearance:

They provide abstractions for most common chart and graph types. They allow the user to customize plots, axes, and legends, both in terms of content (hard-coded or dynamic) and styling (e.g., font type, size, face, and color). For the latter, many provide integrated color palettes, such as Color Brewer. Finally, they solve layout problems given user-provided specifications (such as desired width and height).

### Interactions:

They facilitate interactivity if the deployment method supports it. In many use cases, no interactivity is needed. If it is, then mouse and keyboard allow the user to effectuate interaction techniques, such as highlighting, selecting, and brushing-and-linking.

### Deployment and export:

They export graphics in a variety of vectorized (PDF, Scalable Vector Graphics (SVG), and Encapsulated PostScript (EPS)) and rasterized formats (Portable Network Graphics (PNG), Joint Photographic Experts Group (JPG or JPEG), and Tagged Image File Format (TIFF)) as well as interactive websites (HTML + JavaScript) and allow the user to specify the desired quality if needed. Some enable the embedding of rendered graphics in environments like Jupyter or R Markdown Notebooks.

### Usage by analysts and developers:

They integrate neatly into analytical workflows in support of exploratory analysis (with immediate benefit to the analyst rather than the final audience). They are available for download via standard package repositories, such as Comprehensive R Archive Network (*CRAN*) (R), *pip* (Python), and *npm* (JavaScript). They are available as versioned releases. They often have built-in error reporting to accelerate debugging, with a big community interested in adding new features.

## Existing Work: XR

While some *visualization libraries* exist for *XR* [see this limited overview (GitHub, Zenodo/all Supplemental Tables, Zenodo/direct preview)], they lack critical properties outlined along the five categories for mature 2-D *visualization libraries* listed above.

### Shared pipeline:

Because *XR* visualizations are used outside of traditional WIMP UIs, and only for the visualization part of the workflow, they lack integration with data processing on 2-D screens. While some tools provide a “View in VR” option that supports a *hybrid (work) environment* between a laptop and an *XR device*, it can be challenging to set up, and there is a significant interaction cost to switching between WIMP and *XR* UIs.

### Rendering and appearance:

To the best of the authors’ knowledge, there is no cross-*platform XR visualization library* for use in both *game engines* and WebXR. As a result, many developers build data visualizations from scratch using UI elements, such as sprites, text, buttons, and drop-downs in *game engines*. Another approach is to prebuild visualizations with a 2-D *visualization library*, export them as PNGs or JPGs, and then import them into the *XR* development environment or load them at runtime. However, due to the steps required to integrate 2-D visualizations from established *libraries*, major challenges are the lack of flexibility in this kind of preprocessing and the lack of interactivity. Because the visualizations are prerendered, they are not dynamically changeable or interactable.

### Interactions:

Some engines (e.g., Unreal Engine) provide plug-ins to integrate interactive plots from *plotly* or other *libraries* with HTML export options and project them onto a 2-D plane object within the virtual environment, but these are limited to emulating screen-like user interactivity and constitute less fully featured facsimiles of true 2-D visualizations. This approach also underutilizes natural affordances of *XR*, such as six-degree-of-freedom interaction, spatially distributed views, and colocated multiuser exploration.

### Deployment and export:

While 2-D *visualization libraries* feature many export formats, visualizations built for and in *immersive environments* typically stay there. While both Unity and Unreal Engine offer export to WebGL, the ported code can be difficult to maintain and adjust. A basic method to “take the visualization with you” is to produce a screenshot on the *XR device* with limited resolution and an aspect ratio that may not reflect what the user sees at runtime. Improvements, however, are very much possible. For example, Nanome, a commercial VR tool used for drug design, allows users to take notes alongside screenshots using a dedicated wrist tool.

### Usage by analysts and developers:

Many existing *XR visualization libraries* come as demonstration projects built with *game engines*. Because they are built for (specific versions of) one *platform*, re-using software components elsewhere is difficult or impossible. Porting them or reimplementing them entirely (e.g., from Unity to Unreal Engine) is impractical. Further, documentation is usually lacking and relegated to an associated paper; code is frequently not updated after paper acceptance. As a result, most *XR visualization libraries* have a “snapshot in time” documentation rather than continuously supported product documentation.

Addressing this asymmetry in available *visualization libraries* between 2-D and *XR* requires both bottom–up efforts (e.g., reusable software components and interoperable toolkits) and top–down efforts (e.g., conceptual models, design guidelines, and evaluation *frameworks*) tailored to *immersive analytics*.

## VISUALIZATIONS

**FIGURE 1.**
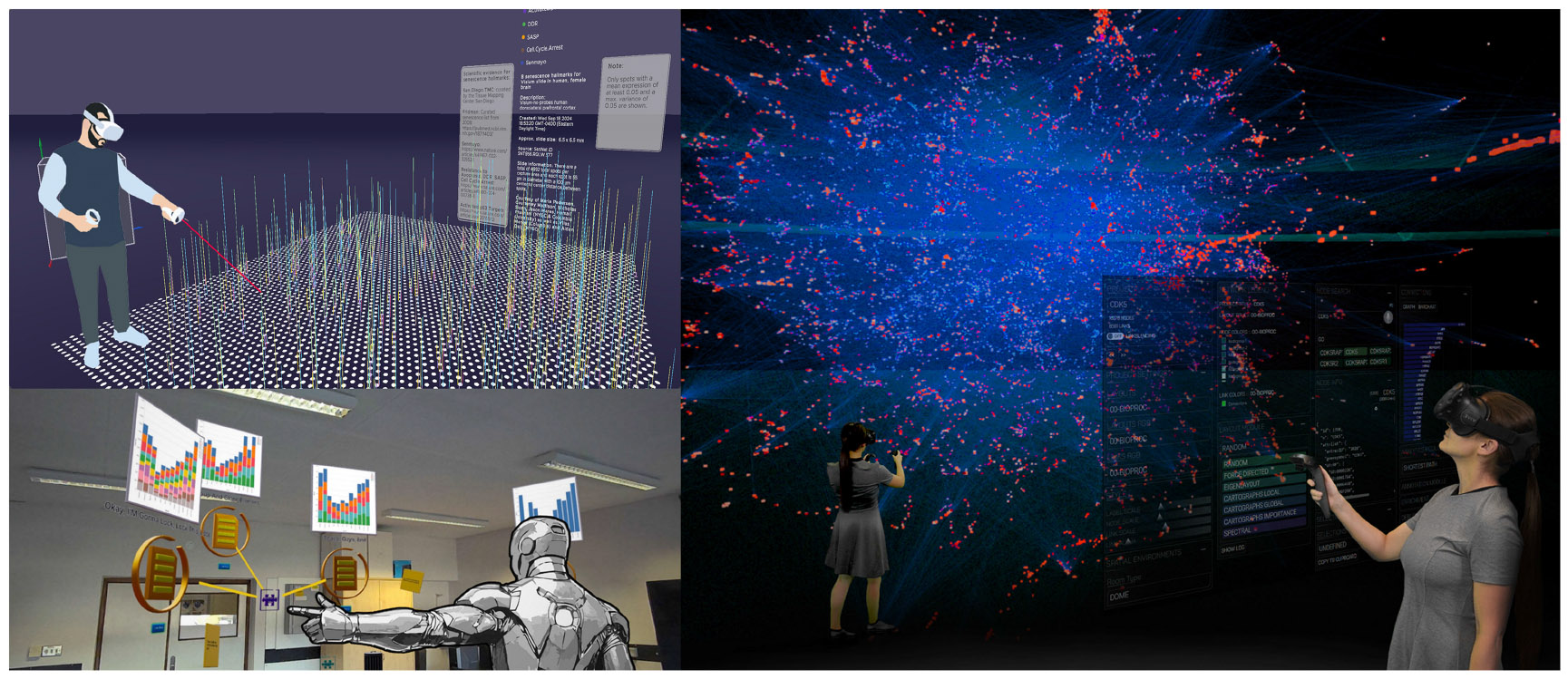
Three applications used during the **TRY** phase of the panel. Top left: HRA Organ Gallery in VR. Bottom left: DashSpace. Right: DataDiVR.

To exemplify different implementations of *immersive analytics*, three applications were shown during the *TRY* phase; see Figure 1. They serve different audiences and use cases and were built with different *platforms* and for different deployment methods: 1) multiscale, biomedical data visualization for 3-D models of adult human organs and harmonized single-cell datasets in the Human Reference Atlas (HRA) Organ Gallery in VR,^8^ built with Unity, running in *stand-alone* mode on consumer-grade Meta Quest headsets; see the HRA Portal and Supplemental Figure 1, 2) collaborative network analysis in DataDiVR,^9^ built with Unreal Engine, running on a VR-ready PC with a tethered *XR device*; see GitHub and Supplemental Figure 2, and 3) DashSpace,^10^ built natively for the web; see GitHub and Supplemental Figure 3. All three applications implement data visualizations differently, and seeing this in action highlighted challenges for creating sustainable, open-source *XR visualization libraries* using data-specific cases. Details are available on GitHub.^g^

## RESULTS

The panel discussion during the *TALK* phase, the hands-on session during the *TRY* phase, and the summarizing exchange during the subsequent *TELL* phase identified four major themes for theory-grounded *XR visualization libraries*.

### Theme 1—Traditional Versus Immersive Visualization

Drawing on practical use cases, attendees argued against fixed categorical rules for 2-D versus 3-D and instead advocated for merging available technologies to develop solutions tailored to specific analytical needs. The discussion should move away from the “horse race” between dimensions toward a focus on functional integration to support seamless and enhanced user experiences. This fits well with a broader view of visualization as a continuum of human-data interaction rather than a set of discrete *interface modalities*. Screen-based, physical, and immersive representations can be seen as successive extensions of how users reason about data through perception, embodiment, action, and space, rather than mutually exclusive paradigms.

#### Heuristics for dimensions:

Determining which visualizations make sense in 2-D or 3-D continues to be a central consideration. While 3-D is beneficial for spatial layout and complexity, viewing and interacting with 2-D visualizations in their natural desktop-native environment remains predominant. Moreover, insights from the field of *data physicalization*, i.e., to represent data with tangible objects in physical (3-D) space, suggest that dimensionality should not only be evaluated in terms of representational capacity, but also in terms of how bodily movement, spatial memory, and manipulation support sense-making in data beyond a natural 2-D/3-D representation.

#### Hybrid interfaces:

To support simultaneous and collaborative 2-D, 3-D, and *embodied exploration*, specific tasks require *hybrid environments* allowing users to communicate their ideas and insights in real-time, as well as to document thought processes through diversified media, from spatial annotations or anchors, to mind-map like drawings, images, and text.

#### Usability as a Toolbox:

Driven by the end users’ need to interconnect diverse data modalities and developers having to prepare data representations for exploration regardless of the increase of complexity, the field is moving toward *scaffold toolboxes*, which act as open, live-reprogrammable environments while accommodating diverse data modalities and allowing for real-time modifications and adaptations ideally within predefined scopes by both users and developers.

### Theme 2—Conceptual Foundations

A shift toward robust, theory-grounded data visualization *frameworks* for *XR* is essential for moving *immersive analytics* into professional practice. Central to this is the adoption of cognitive and *embodied interaction* theories, which provide the foundation for systems where users can interact with data as physical–virtual artifacts.^11^ For instance, lessons from *data physicalization* highlight that material-related constraints play a crucial role in the sense-making process.^12^ While the following foundations primarily guide developers and researchers, they are designed with the end user as the ultimate beneficiary.

#### Existing frameworks :

A point heavily discussed was whether any of the *XR* and *data physicalization* researchers and practitioners were using already existing 2-D-focused *frameworks* when conceptualizing, implementing, and deploying *XR* visualizations, or whether these were insufficient. No consensus emerged, showing the current scattered nature within the field given the broad landscape of tools and practices (developer scope) as well as requirements and expectations (user perspective).

#### Embodied sensemaking:

Based on the lack of existing *frameworks*, we argue for starting with theoretical *frameworks*; these should focus on erasing the boundaries between visualization space and physical reality to allow the user to construct, manipulate, and spatially arrange data representations to fundamentally alter reasoning strategies and long-term retention. The spatial workspace has been shown to act as a primary cognitive resource, where physical interaction within 3-D environments serves as a cognitive tool to enhance the reasoning process beyond the boundaries of *desktop environments*.^13^

#### “Why” of 3-D:

To go beyond simply projecting 2-D charts into the 3-D setting, the community must rigorously evaluate the nontrivial added value of depth and presence to otherwise abstract data representation. This should be grounded in cognitive and *embodied interaction* with the ability to “walk through” the data. Potentially, added value could emerge not from visual novelty, but from enabling new forms of engagement, such as walking around data, comparing viewpoints through movement, or experiencing scale relationally rather than symbolically.^14^

### Theme 3—Tools

The discussion on tools for *immersive analytics* focused on strategic choices between *game engines* and web-based *platforms*,^15^ as well as the limitations of *data physicalization*.

#### Native game engines versus WebXR:

*Native game engines* have traditionally dominated due to their high performance, initial user friendliness for nonprogrammers, and ability to target multiple environments, but they often function as “walled gardens”^15^ with complex update processes, the need for potentially costly licenses, a secluded development system, and lacking integration for widely used open-source tools. Conversely, web-based technologies like WebXR have become increasingly favored based on their interoperability, nonproprietary nature, and ease of deployment via web browsers, which is vital for widespread adoption, but they need to catch up in performance, hardware support, and available components. The accessibility of these web-based tools also addresses significant barriers in developer expertise (see “3-D Skill Gap”).

#### Role of open source:

Open source engines like Godot are gaining traction for cross-*platform* rendering and offer a flexible alternative to proprietary engines for researchers who need to avoid restrictive licensing.

### Theme 4—Challenges to Adoption

Finally, there are systemic and practical challenges to transition *immersive analytics* applications from research prototypes to everyday tools.

#### Demonstrating value:

Often, hardware purchase and setup cost are higher than the hard-to-quantify value that immersive 3-D visualizations offer over traditional 2-D UIs. To move beyond “cool demos,” *XR* visualizations must provide a demonstrable added value over 2-D. Simply projecting 2-D charts into 3-D space is considered a critical pitfall that offers little functional gain.

#### “3-D skill gap”:

A major concern is whether developers must become 3-D experts to translate user’s requirements into applications. By leveraging the WebXR *platform*, one can use existing web development skills instead of having to learn *game engines* with their idiosyncrasies and chosen scripting languages, such as Blueprint and C++ for Unreal Engine, C# for Unity, and GDScript (plus also C#), for Godot.

#### Breaking the habit of 2-D:

Many users are still deeply accustomed to 2-D workflows, which necessitates a focus on smoothing the transitions between the dimensions. This can be achieved by integrating *XR* into daily research based on the balance of cost and benefit for the end users while framing it as an extension of existing analytical practices rather than a replacement.

#### Access and shareability:

A significant barrier is access of hardware/software and expertise required to, e.g., install *stand-alone* applications for *XR devices*. Web-based *platforms* lower this barrier by removing the need for complex installations, allowing anyone with a smartphone or browser to participate in a shared data analysis and visualization. For *stand-alone* applications built with *game engines*, the usage of online storefronts, such as the Meta Store or Steam, where software can be offered for free, can simplify access and installation.

## CONCLUSIONS AND FUTURE DIRECTIONS

In this panel held at IEEE VIS 2025 in Vienna, Austria, on 5 November 2025, *XR* and *data physicalization* researchers discussed key challenges and opportunities for theory-driven data visualization in *XR*. While there was a focus on the (relative lack of) *XR visualization libraries*, the conversation between panelists and audience touched on related, larger issues, such as best use cases and challenges to adoption for *XR* visualization more broadly, resulting in the four themes presented here. The healthy attendance of this panel and lively Q&A session demonstrated the need for continued discussion of how to craft cross-*platform*, scalable, and theory-driven *frameworks* for *XR* data visualizations. The transition of XR from academic novelty to professional practice depends on a shared ecosystem with robust, theory-grounded developer *frameworks* directly relating to and in dialogue with the end users with the goal of insightful, immersive data exploration.

## Figures and Tables

**TABLE 1.** Key terminology for XR visualization.

Term	Definition
Data Physicalization	Representing data in a physical form through, (e.g., rapid prototyping technologies as stand-alone data representation or to enhance/inform digital/virtual environments)
Desktop environment	A computational setup that runs applications on a desktop device only (PC, laptop with 2-D screen)
Embodied exploration	Exploring data in an immersive setting beyond screens while using one’s own body as an input device
XR (XR)	An umbrella term that includes all physical (“real”) and virtual combined environments on a spectrum of experiences, including VR, as being fully immersive and simulated, AR as an overlay of digital components over physical ones, and MR, where realities are blended together to produce new ones. Corresponds to the spectrum of experiences outside of “real environments” in Milgram and Kishino’s reality–virtuality continuum^4^ but also includes VR on the very outside.
Framework	A structured set of principles, rules, and processes that guide the transformation of data into visual representations and explain how those representations convey meaning to an audience. Examples are the Grammar of Graphics^5^ or the Data Visualization Literacy Framework^6^
Grammar of Graphics	A theoretical framework to enable consistent reasoning about visualization design
Hybrid environment	A computational setup that runs applications on a desktop and an immersive device at the same time (e.g., PC VR or WebXR on a desktop with concurrent session in an XR device)
Immersive Analytics	A subdiscipline of information visualization that focuses on theory and practice of using embodied tools to help users understand data and make decisions. Addresses the challenge of today’s data volume and complexity that exceed our ability to comprehend and use it effectively for decision-making. Draws from fields like data visualization, virtual reality, and human-computer interaction to support both individual and collaborative work.^7^
Immersive environment	A computational setup that runs applications on an XR device only (e.g., stand-alone VR)
Interface modalities	UI modes such as immersive, desktop, and hybrid environments
(Native) game engine	A software platform that supplies core tools and systems to create video games; it handles key features, such as graphics, physics, sound, and AI, thus allowing creators to concentrate on designing gameplay and content instead of developing these systems from scratch. Commercial examples are Unity and Unreal Engine; open-source alternatives are Godot, Panda 3D, and Construct 3; and lightweight 2-D/3-D web rendering/game frameworks are Phaser and Babylon.js.
Platform	A code base, like a game engine (e.g., Unity or Unreal Engine), that allows development of XR experiences. A web-based platform is WebXR.
Scaffold toolbox	Open, live, and realtime programmable capabilities of operating an application, applicable to diverse data modalities and shifting from single-purpose to multipurpose usability.
Stand-alone	The headset-only operation mode of an XR device without the need of a PC-powered setup.
Visualization library	A package in a programming language, e.g., an R/JavaScript package, a Python module, or another reusable coding artifact for creating data visualizations, such as *seaborn, ggplot2*, or *Vega-Lite*
XR device	A piece of hardware to experience XR content, [e.g., a head-mounted VR device (Meta Quest, Apple Vision Pro) or AR glasses (HoloLens 2, Magic Leap)]. Many XR devices support multiple interface modalities (hybrid and immersive) and levels of immersion (VR, AR, MR). For example, the Meta Quest headsets and the Apple Vision Pro support VR, AR, and MR applications (although nomenclature for these might differ by manufacturer).

## Footnotes

a [Online]. Available: https://unity.com

b [Online]. Available: https://www.unrealengine.com/en-US

c [Online]. Available: https://immersiveweb.dev

d [Online]. Available: https://cns-iu.github.io/data-vis-xr-panel-2025/#supplemental-table-1-for-visualization-viewpoint

e [Online]. Available: https://zenodo.org/records/19003645

f [Online]. Available: https://zenodo.org/records/19003646/preview/supplemental-table-1.csv

g [Online]. Available: https://cns-iu.github.io/data-vis-xr-panel-2025/#description-of-vr-applications-for-visualization-viewpoint
